# Supplementary material for: Antifouling Systems Based on a Polyhedral Oligomeric Silsesquioxane-Based Hexyl Imidazolium Salt Adsorbed on Copper Nanoparticles Supported on Titania
Source: Nanomaterials (Basel). 2023 Apr 6;13(7):1291. doi: 10.3390/nano13071291 (PMC10096683; doi:10.3390/nano13071291)
Supplement: Supplementary file 1 [file nanomaterials-13-01291-s001.zip › nanomaterials-2319937-supplementary.pdf]

nanomaterials

Article

Antifouling Systems Based on a Polyhedral Oligomeric Silsesquioxane-Based Hexyl Imidazolium Salt Adsorbed on Copper Nanoparticles Supported on Titania

Alessandro Presentato [1], Eleonora La Greca [2], Luca Consentino [1,2], Rosa Alduina [1], Leonarda Francesca Liotta [2,*] and Michelangelo Gruttadauria [1,*]

[1] Dipartimento di Scienze e Tecnologie Biologiche, Chimiche e Farmaceutiche, Viale Delle Scienze, Edificio 17, I-90128 Palermo, Italy; alessandro.presentato@unipa.it (A.P.); luca.consentino@ismn.cnr.it (L.C.); valeria.alduina@unipa.it (R.A.)
[2] Istituto per lo Studio dei Materiali Nanostrutturati (ISMN)-CNR, Via Ugo La Malfa 153, I-90146 Palermo, Italy; eleonora.lagreca@ismn.cnr.it
* Correspondence: leonardafrancesca.liotta@cnr.it (L.F.L.); michelangelo.gruttadauria@unipa.it (M.G.)

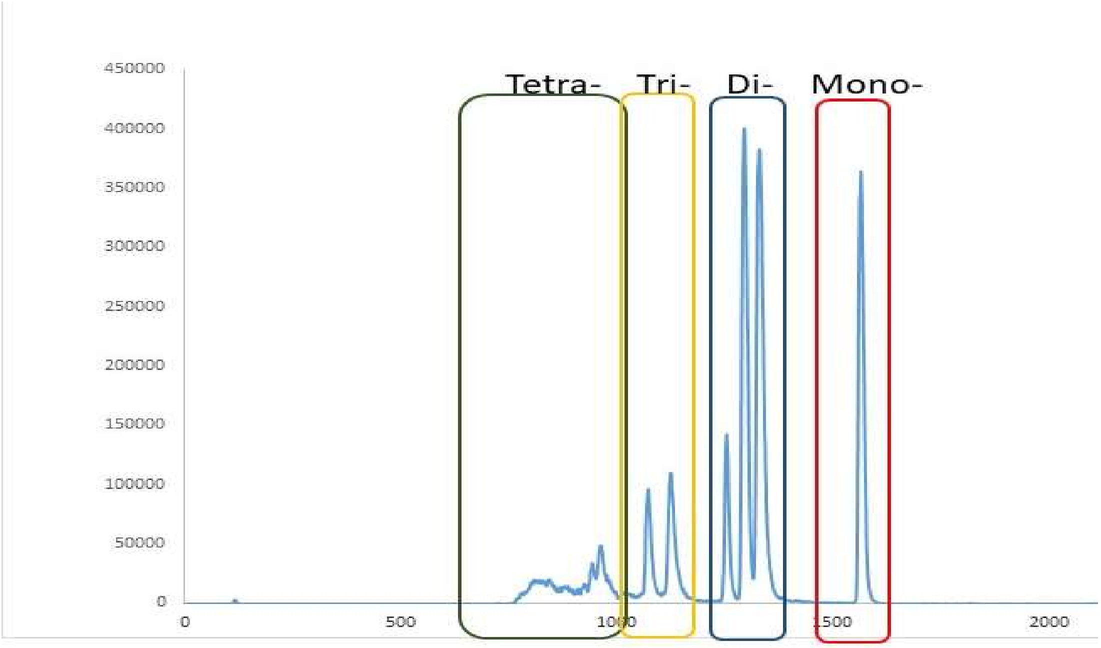

Figure S1. HPLC/MS analysis of **HQ-POSS**.

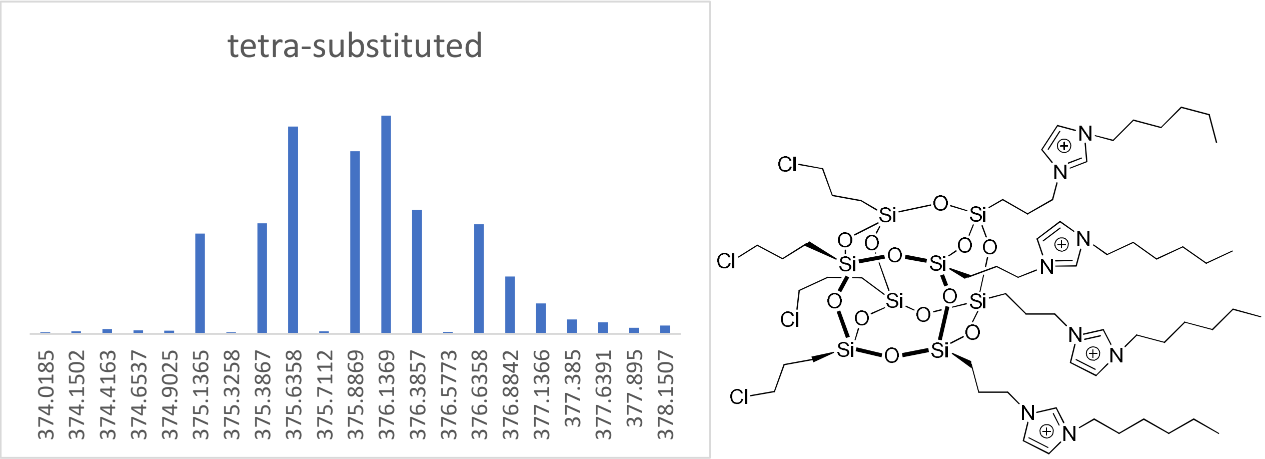

m/z
375.63141

Figure S2. Mass spectrum of tetra-substituted **HQ-POSS**.

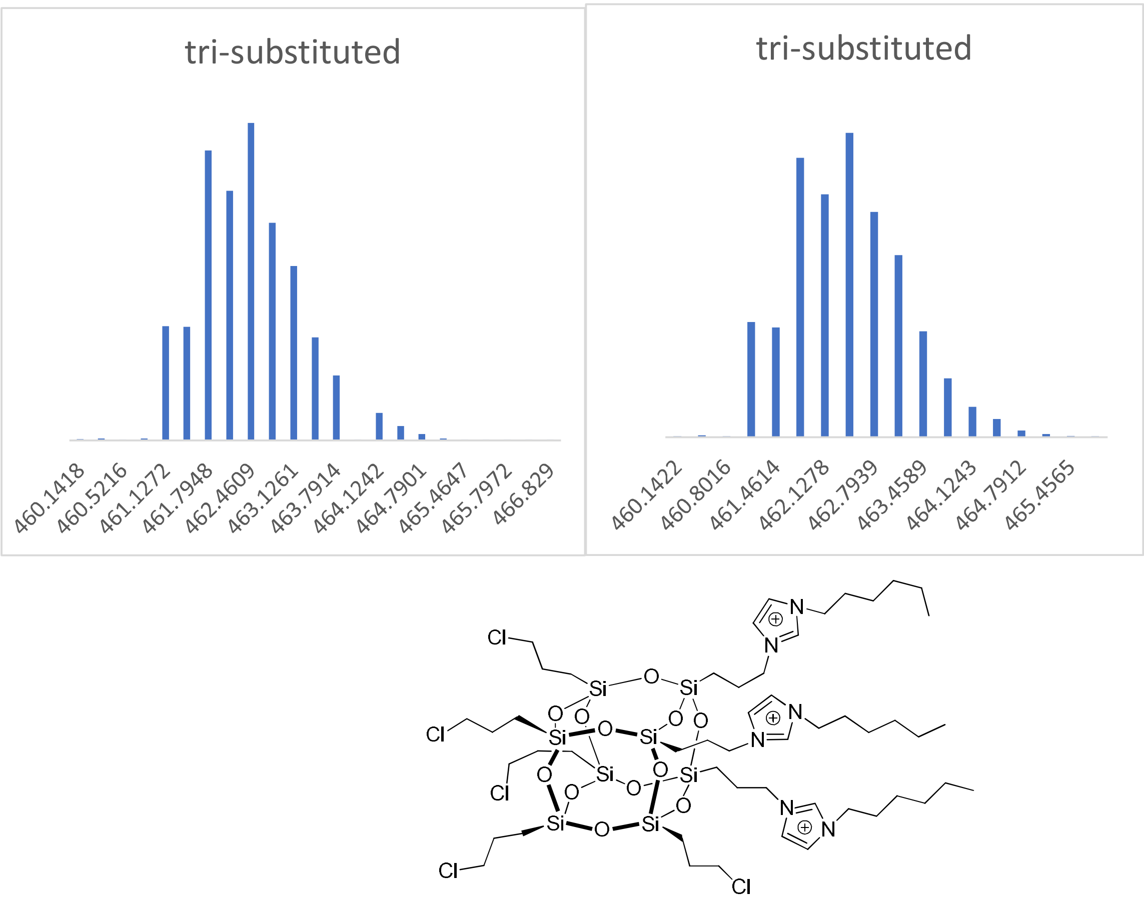

m/z 462.45358

Figure S3. Mass spectra of tri-substituted **HQ-POSS**.

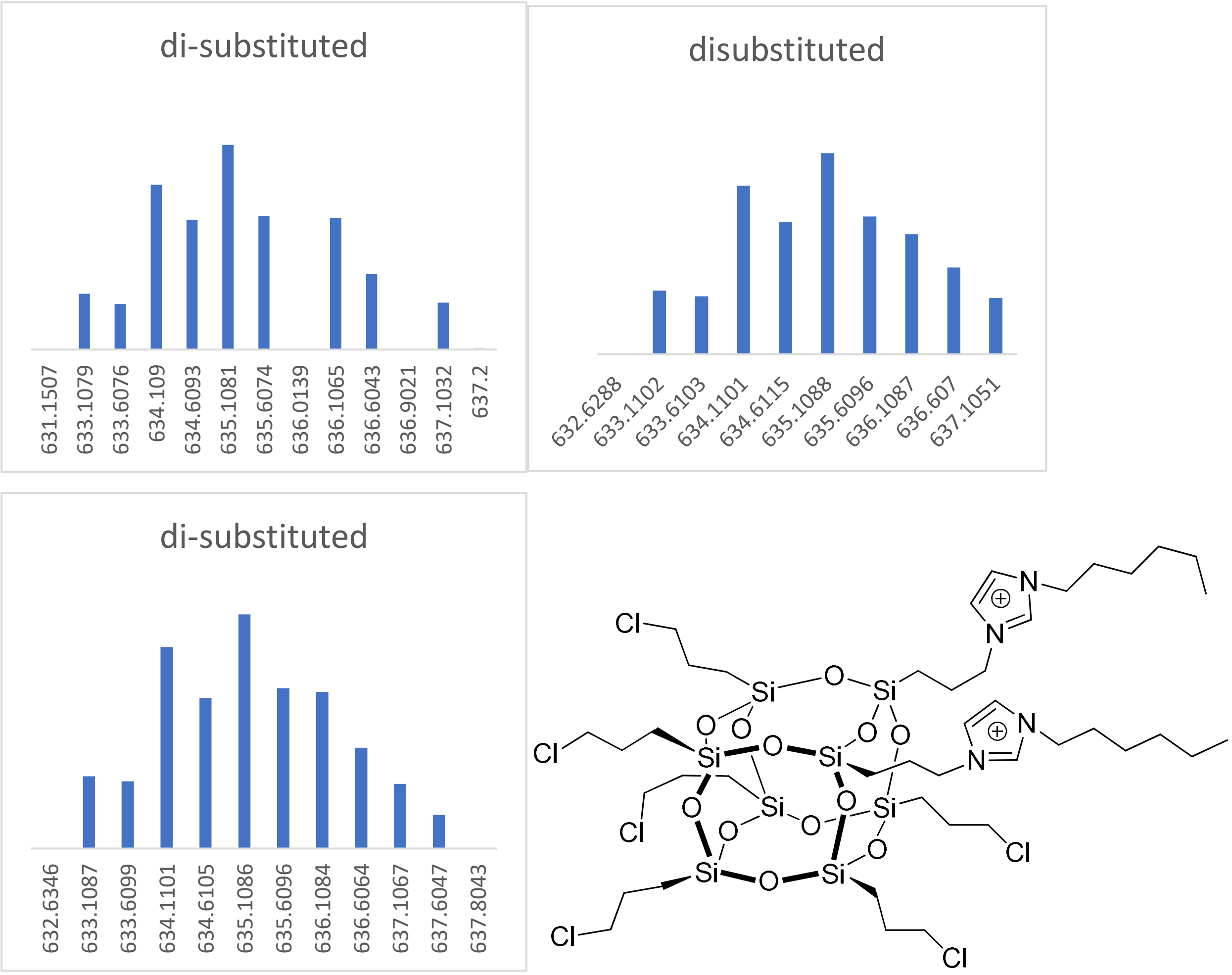

m/z 635.09940

Figure S4. Mass spectra of di-substituted **HQ-POSS**.

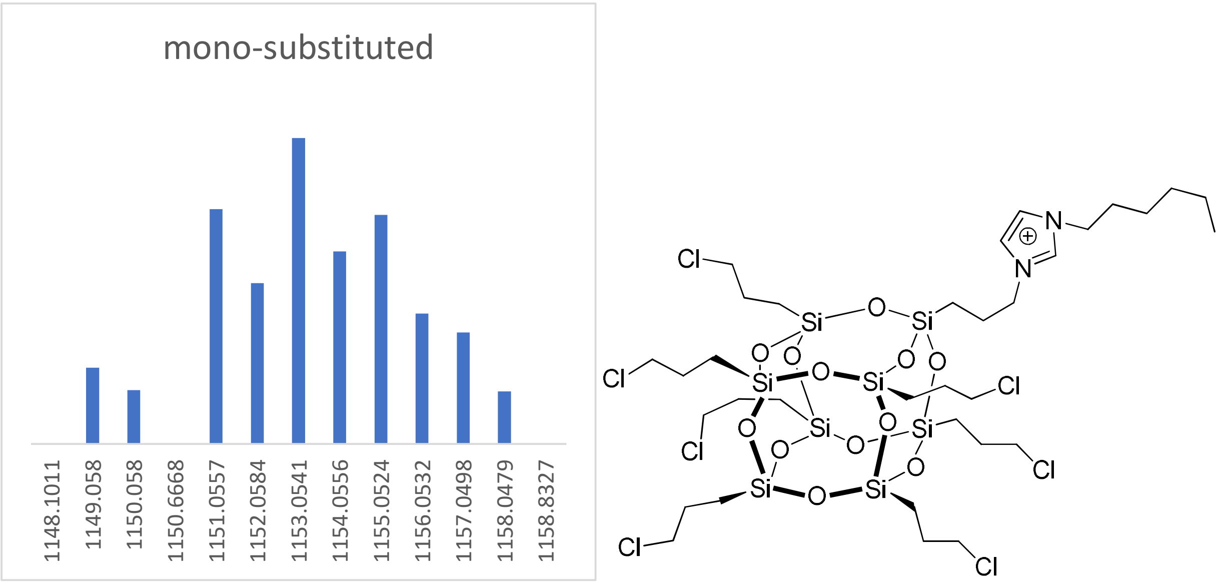

m/z 1151.03981

Figure S5. Mass spectrum of mono-substituted **HQ-POSS**.

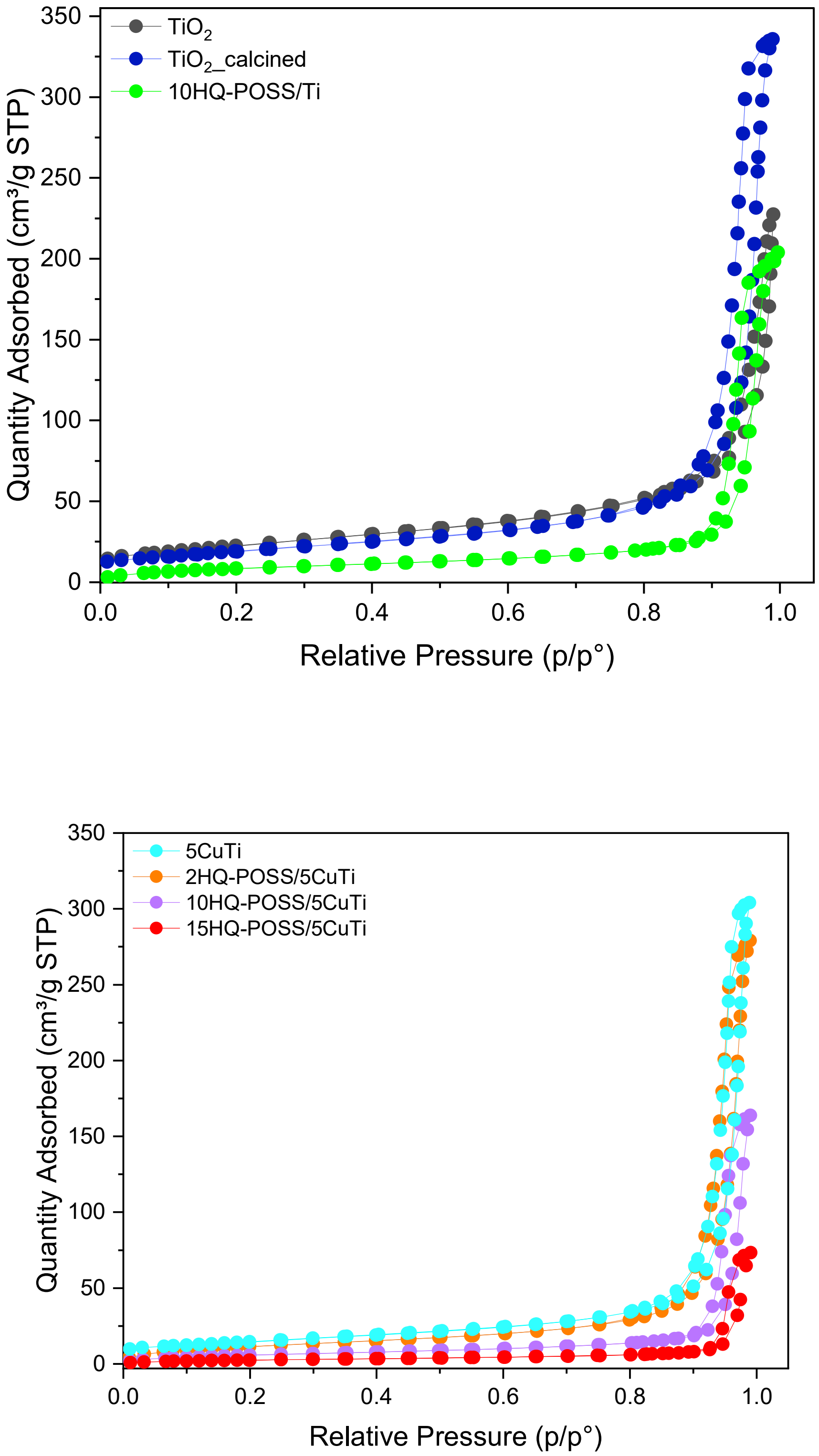

Figure S6. Adsorption-desorption isotherms of samples of **HQ-POSS** and materials.

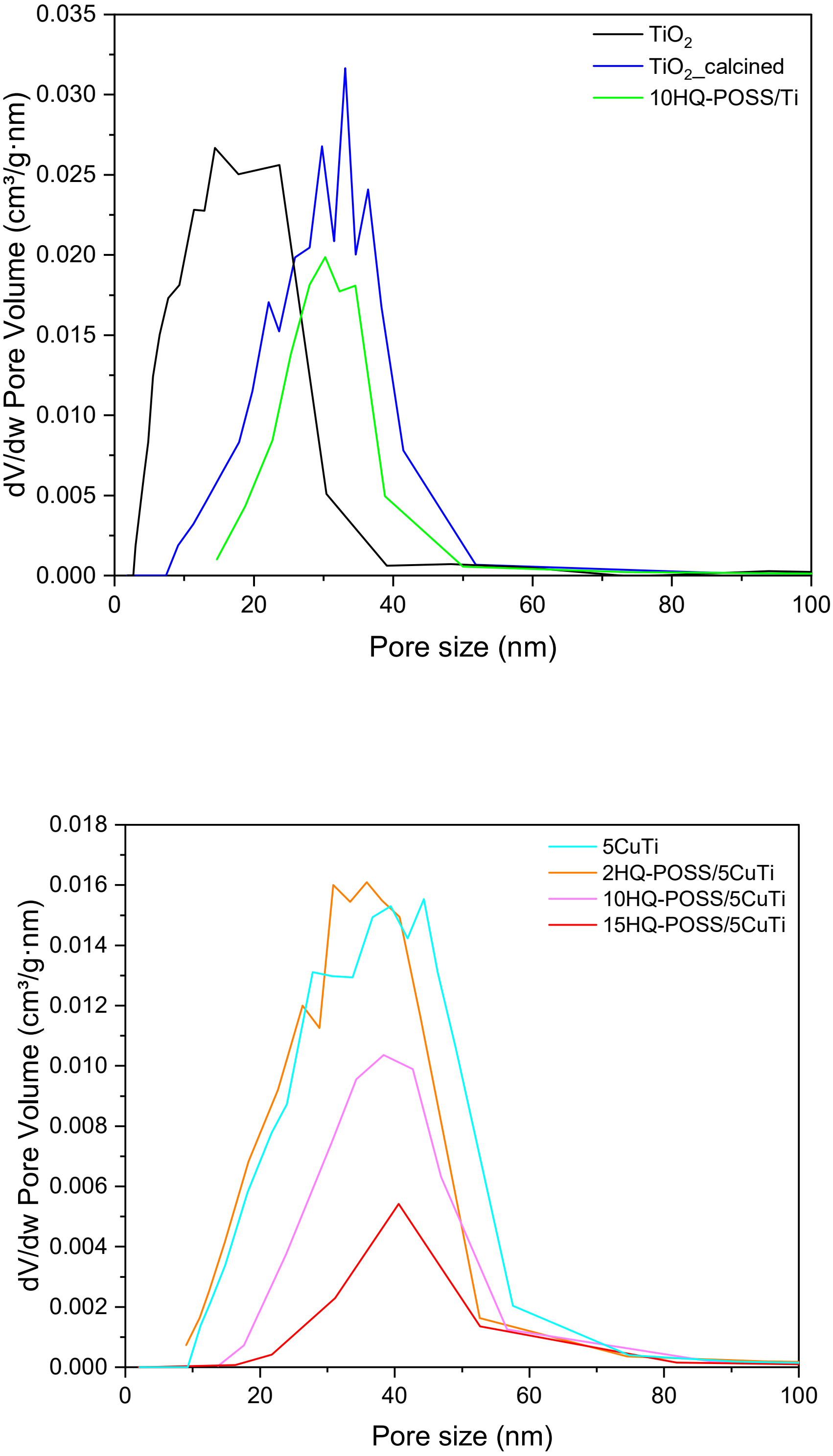

Figure S7. Pore size distribution of samples of HQ-POSS and materials.

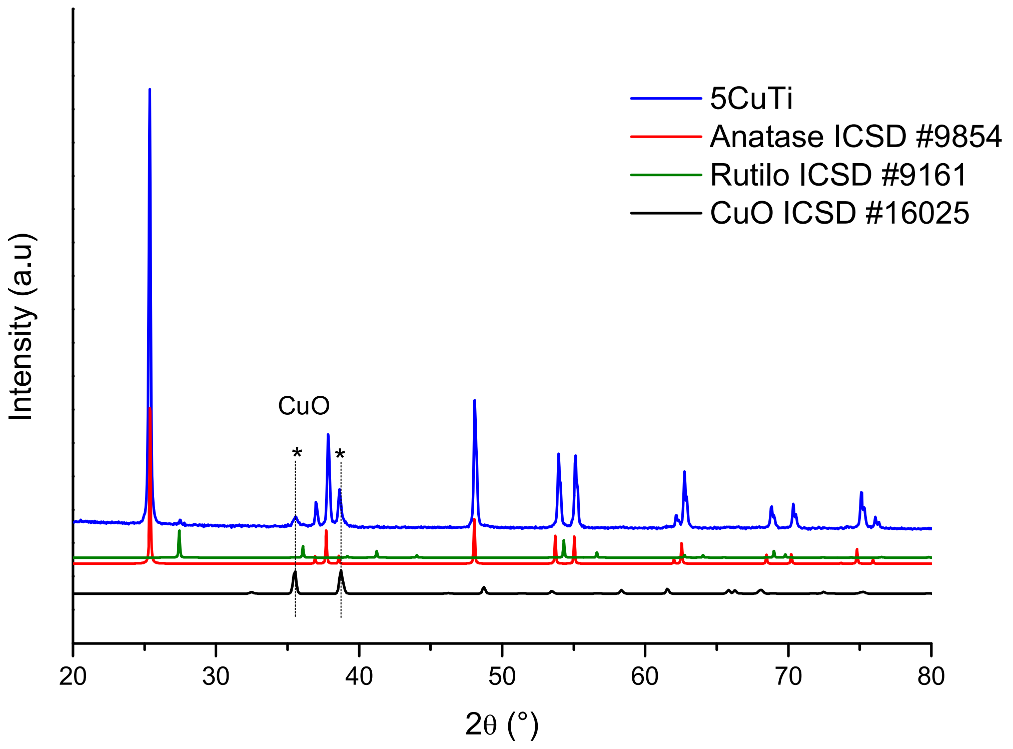

Figure S8. XRD patterns of the material 5CuTi and related reference ICSD files.

Disclaimer/Publisher's Note: The statements, opinions and data contained in all publications are solely those of the individual author(s) and contributor(s) and not of MDPI and/or the editor(s). MDPI and/or the editor(s) disclaim responsibility for any injury to people or property resulting from any ideas, methods, instructions or products referred to in the content.

.
